# Development of Radiotracers for Breast Cancer—The Tumor Microenvironment as an Emerging Target

**DOI:** 10.3390/cells9102334

**Published:** 2020-10-21

**Authors:** Amelie Heesch, Jochen Maurer, Elmar Stickeler, Mohsen Beheshti, Felix M. Mottaghy, Agnieszka Morgenroth

**Affiliations:** 1 Department of Nuclear Medicine, University Hospital Aachen, RWTH Aachen University, 52074 Aachen, Germany; aheesch@ukaachen.de (A.H.); mbeheshti@ukaachen.de (M.B.); fmottaghy@ukaachen.de (F.M.M.); 2 Department of Obstetrics and Gynecology, University Hospital Aachen (UKA), 52074 Aachen, Germany; jmaurer@ukaachen.de (J.M.); estickeler@ukaachen.de (E.S.); 3 Department of Nuclear Medicine, University Hospital Salzburg, Paracelsus Medical University, 5020 Salzburg, Austria; 4 Department of Radiology and Nuclear Medicine, Maastricht University Medical Center (MUMC+), 6202 Maastricht, The Netherlands

**Keywords:** breast cancer, radiotracer, molecular imaging, targeted treatment, tumor microenvironment

## Abstract

Molecular imaging plays an increasingly important role in the diagnosis and treatment of different malignancies. Radiolabeled probes enable the visualization of the primary tumor as well as the metastases and have been also employed in targeted therapy and theranostic approaches. With breast cancer being the most common malignancy in women worldwide it is of special interest to develop novel targeted treatments. However, tumor microenvironment and escape mechanisms often limit their therapeutic potential. Addressing tumor stroma associated targets provides a promising option to inhibit tumor growth and angiogenesis and to disrupt tumor tissue architecture. This review describes recent developments on radiolabeled probes used in diagnosis and treatment of breast cancer especially in triple negative type with the focus on potential targets offered by the tumor microenvironment, like tumor associated macrophages, cancer associated fibroblasts, and endothelial cells.

## 1. Introduction

The multidisciplinary field of nuclear medicine plays an increasing role in oncology. Today, radiolabeled tracers are essential in noninvasive diagnosis, staging, therapy monitoring, and treating different kinds of cancers. A multitude of different radioligands is currently being developed and investigated, each of them uniquely targeting molecular receptors or intracellular components enabling individual patient-tailored therapy planning [1]. For this, radionuclides are coupled to ligands which recognize and bind the tumor associated molecules ensuring the precise targeting of cancerous cells which can be used for both therapeutic approaches and also live-monitoring of the treatment efficacy [2]. The urgent need for tailored approaches yielded development of targeted endogenous radiotherapies as a possible treatment option. This is of particular importance for cancer entities such as breast cancer (BCa) which is characterized by a high degree of heterogeneity in the expression of targetable antigens.

BCa is the most common cancer among women worldwide [3]. The highest incidence is in white women with age being the major risk factor [4,5]. Depending on the type and stage of the cancer, treatment options and prognosis vary significantly. The different subtypes are mainly divided due to the status of receptor expression which serve as biomarkers for optimal treatment [6]. Key players are the estrogen receptor (ER), progesterone receptor (PR), and the human epidermal growth factor receptor 2 (HER2/neu). The numerous different phenotypes highlight the importance of nuclear imaging modalities for accurate diagnosis and personalized treatment options. Imaging with positron emission tomography (PET) or single photon emission computed tomography (SPECT) provides information of the localization of the primary tumor, metastatic situation, as well as the antigen expression level. Besides surgery, BCa patients often receive anthracycline- and taxane-based chemotherapy in combination with radiotherapy. HER2^+^ BCa can be treated with a combined approach of antibody therapy using HER2-targeting antibody trastuzumab and chemotherapy. Steroid receptor positivity in BCa is often treated with endocrine therapy using tamoxifen [7].

About 15% of all BCa lack the expression of ER, PR, and HER2/neu which is considered as triple negative breast cancer (TNBC) [8]. This very aggressive form has a significantly earlier age of onset than other types. Furthermore, at the time of diagnosis, the tumor is larger in size and higher-graded comparing to the other BCa types [9]. This phenotype usually shows a poorly differentiated histology and tends to develop more frequently lymph node metastases with a higher affinity for lung and brain metastases [9,10]. The heterogeneity of TNBC is reflected in the six molecular subtypes which include two basal-like, an immunomodulatory, a mesenchymal, a mesenchymal stem-like, and a luminal androgen receptor subtype. Each of them exhibits unique characteristics like altered biological pathways, genetic mutations, and different cell types [11]. Current clinical treatments of TNBC include cytotoxic chemotherapy, radiation, and surgery [8]. As these options are not targeted, the efficacy is fairly limited and the overall prognosis for TNBC patients is relatively poor [12,13]. To improve the long-term prospects for patients it is necessary to precisely analyze and understand the origin and progression of this disease on a molecular and cellular level.

Lacking all three receptors, which serve as targets in the treatment of receptor expressing phenotypes, makes it more difficult to directly target TNBC cells. Analyzing the tumor microenvironment of TNBC lead to the identification of new targets for prognosis and guided therapies especially as it relates to invasive and metastatic progression [14,15]. The breast environment consists of an extracellular matrix (ECM) and a variation of different cell types such as epithelial cells, endothelial cells (ECs), immune cells, fibroblasts, and adipocytes which are necessary to maintain the structure and function of the breast. The transition from normal to cancerous structures is accompanied by epigenetic and morphological changes of luminal and myoepithelial cells, enhanced angiogenesis, activated fibroblasts, and an increase in infiltrating leukocytes. During these processes some cells undergo an epithelial to mesenchymal transition (EMT). The acquisition of mesenchymal features enables them to invade tissues and migrate to distant sites [16]. Through the release of soluble mediators like cytokines tumor cells are able to communicate with adjacent cell types like tumor-associated macrophages (TAMs), cancer-associates fibroblasts (CAFs), and ECs. Some factors have pro-angiogenic effects which include vascular endothelial growth factor (VEGF), fibroblast growth factors (FGFs), hepatocyte growth factor (HGF), platelet derived growth factor (PDGF), monocyte chemoattractant protein (MCP)-1, macrophage inflammatory protein (MIP)-1β, interleukin (IL)-8, and CC-chemokine ligand 5 (CCL5) [17]. Chemosensory reception of these factors manipulates the cells into a pro-tumoral state supporting carcinogenesis [14,18]. Overexpressed receptors on the surface of these cellular compartments present potential targets to destruct tumor tissue, and to inhibit tumor growth and angiogenesis.

**Figure 1:**
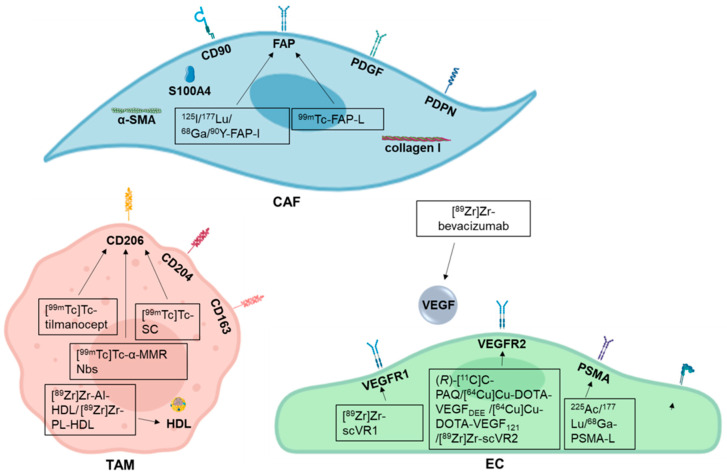
Breast cancer microenvironment targets and their respective radiotracers addressing cancer-associated fibroblasts (CAFs), tumor-associated macrophages (TAMs), and endothelial cells (ECs) discussed in this review. The black boxes display the available targeting agents that are currently object of research. -I: inhibitor; -L: ligand.

In this review we focus on three key players of the tumor microenvironment, CAFs, TAMs, and ECs which offer several targets for guided approaches (Figure 1).

We also describe different radiotracers and their potential for diagnosis and radiotherapies of BCa with particular focus on TNBC. The tracers discussed in this review are summarized in Table 1.

## 2. The Importance of Nuclear Medicine in Cancer Management

**Figure 2:**
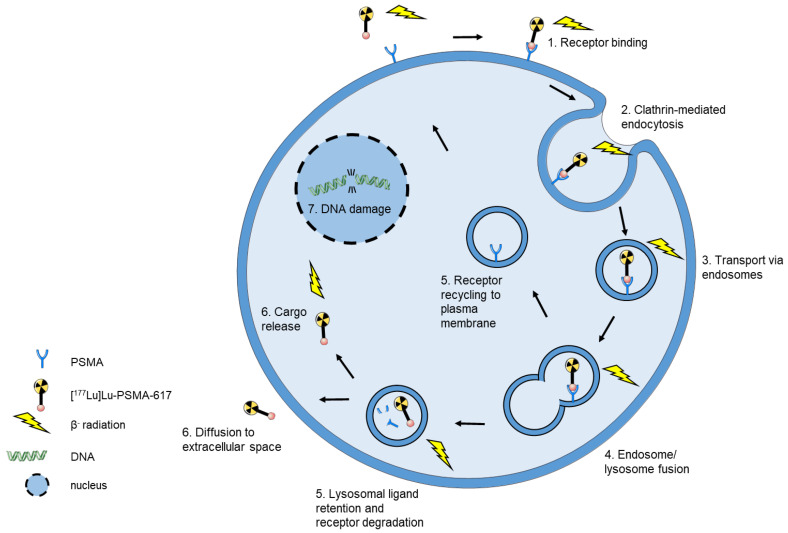
Schematic model of [^177^Lu]Lu-PSMA-617 internalization in endogenous radiotherapy. (**1**) Upon binding of [^177^Lu]Lu-PSMA-617 to PSMA (**2**) the complex is internalized into the tumor cell via clathrin-mediated endocytosis. (**3**) The tracer is transported throughout the cell via endosomes which (**4**) fuse with a lysosome. (**5**) PSMA is recycled back to the plasma membrane or undergoes lysosomal degradation. (**6**) The tracer is released or the ligand may diffuse to the extracellular space [55]. Since the β-rays of ^177^Lu have an average soft tissue range of 0.23 mm which surpasses the cell diameter, an internalization of [^177^Lu]Lu-PSMA-617 is not necessary for the cell toxicity. Importantly, this enables destroying of surrounding cancer cells which do not express the target (crossfire effect addressing tumor heterogeneity) [56].

The application of radionuclides in modern medicine opened new possibilities for the imaging and therapy of primary and metastatic cancers. Theranostic approaches combine two different radionuclides, one for imaging and one for therapy. Depending on diagnostic or therapeutic application, the respective radionuclide is coupled to the suitable ligand. A common pair for theranostic application is ^68^Ga and ^177^Lu coupled to PSMA-617 which are currently used in diagnostics and therapy of prostate cancer (PCa). [^68^Ga]Ga-PSMA-617 is used to image tumor uptake and monitors the efficacy of the treatment whereas [^177^Lu]Lu-PSMA-617 serves as the therapeutic equivalent. ^177^Lu is frequently used for endogenous radiotherapy because it possesses an advantageous combination of energy deposition and physical half-life compared to ^90^Y. Due to the lower range of tissue penetration, ^177^Lu causes less damage to adjacent normal tissue and renal toxicity is significantly lower for ^177^Lu in comparison to ^90^Y [51,52]. ^177^Lu emitted β^−^ particles have a range of average 0.23 mm (max. 1.7 mm) in soft tissue which is ideal to deliver energy to small volumes such as micrometastases, whereas the average penetration depth of ^90^Y β^−^ particles in human tissue is 2.4 mm and is of advantage in larger tumors [53,54]. The half-life of 6.65 days allows labeling of peptides with slower targeting kinetics and minimizes decay loss during transportation procedures [53]. [^177^Lu]Lu-PSMA-617 is intravenously injected into the patient and subsequently delivered to its molecular target via the blood stream. The delivery of [^177^Lu]Lu-PSMA-617 for endogenous radiotherapy is illustrated in Figure 2.

## 3. Direct Targeting of Tumor Cells

Making the right diagnosis is essential for the individual treatment choice. Anatomical imaging options for the detection of BCa include mammography (MM), ultrasound (US), computed tomography (CT) and magnetic resonance imaging (MRI). With the molecular imaging approaches using radionuclides detectable in PET or SPECT it is possible to visualize also specific biological processes. Moreover, by using radiotracers addressing a receptor, a protein or another biological compound radionuclide imaging offers a more specific targeting of the tumor tissue [57].

### 3.1. ER^+^ BCa

ER-positivity applies for ≈80% of all BCa [58]. One of the most extensively studied radiotracers to image ER^+^ BCa is 16α-[^18^F]fluoro-17β-estradiol (FES). Estradiol acts as an agonist for the ER enabling the binding of the radiotracer. Due to its high binding affinity, [^18^F]FES-PET provides clear images of primary and metastatic BCa [59]. In a previous study, the [^18^F]FES uptake of the tumor was shown to correlate with ER expression as determined by immunohistochemistry (IHC) [60,61]. Additionally, [^18^F]FES-PET predicts effectiveness of endocrine therapy. As demonstrated by several studies, [^18^F]FES-PET visualizes clinical benefits of ER-directed treatment options for the individual patient and provides diagnostic information [60]. Unfortunately, FES is rapidly metabolized in the liver into sulfate and glucuronate conjugates which are unlikely to bind to ER-expressing cells resulting in a high image background [62,63]. Moreover, the hepatic uptake and biliary excretion of this tracer additionally complicate the detection of lesions in this region. FES also shows affinity to plasma globulins which results in a reduced amount of tracer available for targeting the ER receptors expressing cells [19]. A modification of the estradiol to 4-fluoro-11β-methoxy-16α-[^18^F]FES ([^18^F]4FMFES) lowers tracer affinity for plasmatic globulins, accelerates blood clearance, and decreases uptake in nonspecific organs compared to [^18^F]FES [19,64]. In a Phase II clinical trial, the diagnostic potential of [^18^F]4FMFES was further evaluated. The tracer showed a similar tumor targeting potential to [^18^F]FES but had superior imaging properties due to decreased overall background allowing higher detection rates and better sensitivity towards ER^+^ BCa [19].

### 3.2. PR^+^ BCa

For PR^+^ BCa, which accounts for ≈65–75% of all BCa [65], imaging agents include 21-[^18^F]fluoro-16α-ethyl-19-norprogesterone ([^18^F]FENP) and 1-[^18^F]fluoro-16α,17α-[(R)-(1′-α-furylmethylidene)dioxy]-19-norpregn-4-ene-3,20-dione ([^18^F]FFNP). [^18^F]FENP was the first studied PET-radiotracer for imaging of PR expression. Although it has shown high binding affinity for the PR in rodents, in patients the tracer was able to identify only 50% of PR^+^ tumors correctly. The tracer uptake did not correlate with PR expression levels in the tumors and the overall target/background ratio was very low which together resulted in clinical application failure [66,67]. [^18^F]FFNP is more promising as it has a higher binding affinity to PR and less unspecific binding [68]. An ongoing Phase II study is evaluating [^18^F]FFNP-PET/MRI for imaging of PR expression in invasive BCa patients (NCT03212170).

### 3.3. HER^+^ BCa

The third key player is HER2/neu which is amplified in ≈18–20% of BCa [69]. Different humanized radiolabeled antibodies are available to target HER2/neu. The most studied is trastuzumab, a humanized murine monoclonal antibody which inhibits the growth of HER2/neu overexpressing human cancer cells [70]. Perik et al. showed that [^111^In]In-trastuzumab is able to identify HER2^+^ tumors via SPECT but its potential is limited due to the spatial resolution [71].

An early Phase I study evaluating [^89^Zr]Zr-trastuzumab for PET imaging in 12 female HER2^+^ BCa patients (six of which had primary and six had metastatic BCa) showed an excellent tumor delineation of tumor lesions and no adverse effects. Best PET image quality was obtained after 4 and 6 days after tracer administration. Overall, PET imaging of HER2 expression with [^89^Zr]Zr-trastuzumab is safe with an acceptable dosimetry. However, a limitation of this radiotracer is its high accumulation in the liver [72].

A different radioligand, [^64^Cu]Cu-DOTA-trastuzumab, was evaluated regarding tumor uptake in women with metastatic BCa. The group was subdivided in HER2^+^ and HER2^−^ patients but all patients had at least low-level expression (IHC1+) of HER2 in a biopsied tumor. Uptake of [^64^Cu]Cu-DOTA-trastuzumab was higher in HER2^+^ and increased tumor uptake was observed between days 1 and 2 for both groups. Moreover, the tumor uptake correlated with the patient HER2 status [73].

Although radiolabeled trastuzumab has been demonstrated as a useful tool for HER2-targeted imaging, a further more specific antibody, pertuzumab, has been developed. [^89^Zr]Zr-pertuzumab accumulated specifically in HER2^+^ BT474 xenograft which was enhanced by prior injection of unlabeled trastuzumab. This effect is due to conformational change of HER2 induced by trastuzumab binding which leads to a higher exposure of the pertuzumab binding site. The tracer demonstrated high affinity and high selectivity for HER2 but also high variance in tumor uptake which is probably due to tumor growth variability [74]. In 2018, the first-in-human study with [^89^Zr]Zr-pertuzumab for PET/CT imaging in patients with biopsy-proven HER2^+^ malignancies was published. The biodistribution and normal tissue dosimetry of [^89^Zr]Zr-pertuzumab were comparable with those of [^89^Zr]Zr-trastuzumab. A big advantage was that HER2 targeted therapy did not have an impact on [^89^Zr]Zr-pertuzumab binding and PET imaging [75].

Recently, the use of HER2 targeting antibody fragments gained attention. The small size allows better tumor penetration, lower immunogenicity, and cheaper and larger scale production compared to full length antibodies [76]. In a Phase I study, [^68^Ga]Ga-HER2-Nanobody was evaluated for PET imaging of HER2 expressing tumors in 20 women with primary or metastatic HER2^+^ BCa (IHC proven). The unbound tracer was rapidly cleared from the blood allowing imaging at early time points (60–90 min post injection). Renal excretion resulted in the highest organ dose in the urinary bladder wall. Uptake in primary lesions varied widely but in patients with metastatic disease, distinct uptake was seen in most metastases. Thus, the tracer may be better suited for visualization of metastatic stages than of primary lesions [21].

Another Phase I/II study evaluated the potential of [^68^Ga]Ga-ABY-025 for PET imaging of HER2 expressing cancer. The ligand ABY-025 is an affibody molecule which specifically binds to a different HER2 epitope than current HER2 targeting therapeutic drugs. Subsequent imaging (4 h post-injection) visualized HER2^+^ metastases preferably. The uptake in HER2^+^ lesions was 5-fold higher than in HER2^−^ lesions and PET SUV correlated with IHC scores. Together, [^68^Ga]Ga-ABY-025 PET/CT accurately identified HER2 expression in BCa metastases [77].

A further HER2 addressing nanobody [^18^F]F-RL-I-5F7 demonstrated rapid tumor accumulation and blood clearance in a BT474M1 xenografted mice model. Pretreatment with trastuzumab decreased tracer binding more than 10-fold confirming HER2-specificity. Importantly, [^18^F]F-RL-I-5F7 shows a high renal clearance rate, and no retention in the liver, a frequent site of metastases for HER2^+^ BCa [22].

[^18^F]F-RL-I-2Rs15d is a radiolabeled single-domain antibody targeting HER2. It binds to a different epitope of HER2 which enables monitoring of therapy with trastuzumab or pertuzumab. [^18^F]F-RL-I-2Rs15d was shown to detect HER2^+^ brain lesions proving the ability of this tracer to cross the blood–brain barrier. However, [^18^F]F-RL-I-2Rs15d exhibited lower tumor cell retention both in vitro and in vivo than 5F7 therefore further optimization is required [23].

Additionally to diagnostic application some antibody fragments have been developed and evaluated as theranostic tools for radio immunotherapy (RIT), for example [^131^I]I-SGMIB-2Rs15, a HER2 addressing single-domain antibody. SPECT imaging visualized HER2 specific uptake in BT474M1 tumor and very low accumulation was observed in thyroid and muscle. In a therapy study, [^131^I]I-SGMIB-2Rs15 alone or combined with trastuzumab significantly extended the median survival of BT474M1 mice [24].

For alpha emitter therapeutic approach the 2Rs15d nanobody was labeled with ^225^Ac ([^225^Ac]Ac-DOTA-Nb). In vitro, the viability of SKOV-3 ovarian cells (overexpressing HER2) decreased rapidly with increasing doses of [^225^Ac]Ac-DOTA-Nb. Binding of [^225^Ac]Ac-DOTA-Nb to MDA-MB-231 cells was 60–70 times lower compared to SCOV-3 cells. Tumor uptake of [^225^Ac]Ac-DOTA-Nb ex vivo was about eight times higher in SCOV-3 xenografts than in MDA-MB-231 xenografts. [25].

A recent study evaluated the potential of 2Rs15 labeled with ^111^In or ^225^Ac for RIT of HER2^+^ brain metastases. [^111^In]In-2Rs15d showed low uptake in normal tissue and high specific uptake in HER2^+^ brain tumor. Therapy with [^111^In]In-2Rs15d and [^225^Ac]Ac-2Rs15d resulted in prolonged survival of animals treated with [^111^In]In-2Rs15d or [^225^Ac]Ac-2Rs15d alone or combined with trastuzumab when compared to monotherapy with trastuzumab or buffer control. These results demonstrate 2Rs15d as a valuable vehicle for a theranostic approach in detecting and therapy of HER2 expressing brain metastases of BCa [26].

The general problem of these applications, the renal toxicity, was addressed by an approach with affibody-based peptide nucleic acid (PNA)-mediated pretargeted therapy. For this, an affibody molecule was conjugated with an AGTCGTGATGTAGTC PNA hybridization probe (Z_HER2:342_-SR-HP1) as the primary pretargeting agent. A complementary AGTCGTGATGTAGTC PNA conjugated to DOTA labeled with ^177^Lu ([^177^Lu]Lu-HP2) was used as the secondary agent. After pretargeted treatment, the absorbed dose in tumors was 5-fold higher than in kidneys. Despite the potential to extend survival of SCOV-3 mice, the renal uptake was still problematic when applying the same dose of [^177^Lu]Lu-HP2 [27].

### 3.4. TNBC

Unlike the above described BCa phenotypes, TNBC lacks the receptors which are essential for targeting approaches. Nevertheless, research is progressing towards identification of novel targets and development of imaging and theranostic agents for treatment of TNBC.

One of them is urokinase plasminogen activator receptor (uPAR), which has been shown to be overexpressed in BCa, associated with aggressive tumor behavior and acquired drug-resistance. In a targeted approach uPAR as an imaging target was evaluated in BCa models of acquired drug resistance. SPECT/CT analysis with the ^111^In-labeled anti-uPAR antibody 2g10 demonstrated high tumor uptake in uPAR expressing xenografts. No uptake was observed in the uPAR^−^ tumor. Moreover, the tracer uptake was increased in a drug resistant tumor model. This study indicated that uPAR could serve as a useful marker of acquired drug resistance in BCa especially in TNBC [28].

Another protein which is upregulated in different cancers including TNBC is tissue factor (TF) which is associated with an increased morbidity in these patients. Shi et al. generated a Fab fragment ALT-836-Fab which was labeled with ^64^Cu. PET images revealed rapid uptake and retention of [^64^Cu]Cu-NOTA-ALT-836-Fab in MDA-MB-231 xenografts with high tumor to background ratio. A limitation of this approach is the lower binding avidity of Fab fragments which is problematic if high absolute tumor uptake rather than high tumor contrast is required [29].

Another approach addresses the high expression of glycoprotein non-metastatic B (gpNMB) in TNBC as a target for PET imaging. Glembatumumab vedotin (CDX-011) is an antibody-drug conjugate which targets gpNMB. In this study, the antibody glembatumumab (CR011) labeled with ^89^Zr ([^89^Zr]Zr-DFO-CR011) was evaluated for selection of patients responding to targeted treatment with CDX-011. [^89^Zr]Zr-DFO-CR011 bound specifically to gpNMP in vitro and in vivo. Moreover, this tracer has potential to visualize different levels of cell surface gpNMB expression on tumor cells. However, selective targeting of gpNMB in the tumor versus in normal organs could not be assessed in the animal study, since the CR011 antibody binds selectively to human gpNMB [30].

Folate receptor α (FRα) is overexpressed on TNBC cells and therefore can also serve as a potential biomarker. In a recent study, ^89^Zr-radiolabeled M9346A antibody against FRα was evaluated in vitro and in vivo. Cell binding assays revealed a 20-fold higher binding of [^89^Zr]Zr-M9346A to high FR expressing tumor cells. In vivo, significant tumor uptake was observed 72 h post administration. In TNBC patient-derived xenograft (PDX) models, the tumor uptake of [^89^Zr]Zr-M9346A correlated well with RT-PCR expression analysis. These results indicate the potential of [^89^Zr]Zr-M9346A as in vivo diagnostic [31].

Targeting alkylphosholipid derivatives which mimic lipid rafts in cell membranes can be a useful theranostic approach as these are markedly overexpressed in malignant cells. ^177^Lu-labeled alkylphosphocholine (NM600) was investigated for therapeutic use in TNBC. The dosimetry PET study using [^86^Y]Y-NM600 in TNBC xenografted models visualized a favorable tracer biodistribution. In the therapeutic study, the absorbed tumor doses resulted in effective tumor growth inhibition and extended survival compared to controls. In the non-metastatic TNBC model, complete tumor eradication was achieved in 50–60% of the treated animals. In the metastatic TNBC model, [^177^Lu]Lu-NM600 was not able to completely stop metastatic progression. This might be due to the incomplete formation of vasculature of the metastases resulting in a limited availability and uptake of [^177^Lu]Lu-NM600. In future studies, therapeutic potential of high linear energy transfer (LET) radionuclides like ^225^Ac, ^227^Th or ^212^Pb will be evaluated for treatment of metastatic TNBC [32].

Currently, Poly ADP ribose polymerase (PARP) is intensively studied as a potential BCa target. Mutated *BRCA* is associated with sensitivity to PARP inhibition which raised interest for application of PARP inhibitors in breast and ovarian cancers. Based on this the core structures of PARP inhibitors have been used for development of PARP addressing tracers. [^18^F]fluorthanatrace (FTT) is the first PARP-targeted radiotracer which has been tested in humans. In vitro, the cellular uptake of [^18^F]FTT correlated with PARP1 expression. In TNBC xenografted models, the PET imaging visualized PARP expressing tumor with a T/B ratio of 1.9 in HCC1937 xenografts and 1.5 in MDA-MB-231 xenografts. Moreover, the pretreatment with the PARP-inhibitor blocked tumor uptake of [^18^F]FTT [33]. Promising in vitro and in vivo results lead to several in-human trials in primary or recurrent BCa which are currently ongoing and are expected to be completed in 2023 (NCT03846167, NCT03083288, NCT03604315). Another PARP-targeting agent is I2-PARPi labeled with either [^131^I] (SPECT) or [^124^I] (PET) developed for glioblastoma imaging. Both imaging modalities succeeded to visualize the tumor with specific uptake in the brain hemisphere where the xenograft was implanted. Ex vivo autoradiography supported the in vivo results. Olaparib treatment strongly decreased the signal intensity of [^131^I]I2-PARPi proving the specificity of the tracer. In summary, the results highlight the specificity and potential of this tracer for glioblastoma imaging [34]. As a theranostic approach, PARPi can be labeled with Auger emitters which was evaluated in a recent study. Meitner-Auger PARPi (MAPi) was labeled with [^123^I] for small-animal SPECT/CT. Orthotopic xenografts of TS543 cells were implanted in one brain hemisphere. Treatment with [^123^I]I-MAPi was delivered at week 3. Full-body images showed retention of [^123^I]I-MAPi in the glioblastoma 18h post-injection. Further therapy monitoring confirmed the efficacy of the theranostic agent which reflected in significantly improved survival for the [^123^I]I-MAPi treatment cohort compared to the control. Together, this study illustrates the importance and potential of the first Auger-based theranostic PARPi for future application in PARP-targeted radiotherapy [35].

## 4. Targeting of the Tumor Microenvironment

Since currently used agents targeting HER2, PR, and ER are ineffective for the treatment of TNBC, different options need to be developed and evaluated. The tumor microenvironment of BCa presents several valuable targets and therefore is an excellent candidate to bypass a direct tumor cell targeting approach.

**Figure 3:**
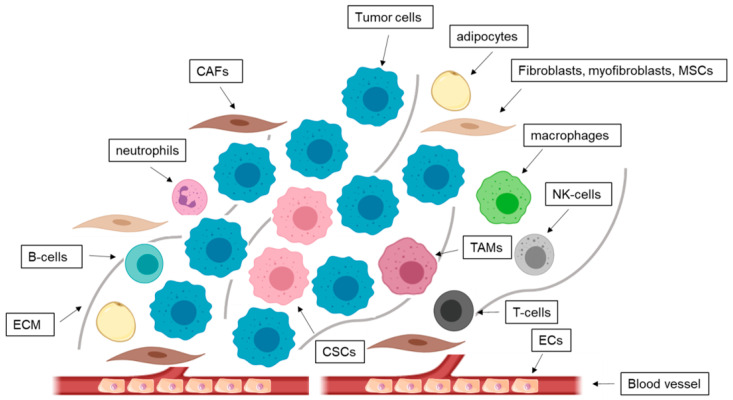
Microenvironment in breast cancer (BCa). The tumor microenvironment consists of numerous cell types like fibroblasts, myofibroblasts, mesenchymal stem cells (MSCs), cancer-associated fibroblasts (CAFs), adipocytes, endothelial cells (ECs), and immune cells like T-cells, B-cells, natural killer (NK) cells, macrophages, tumor associated macrophages (TAMs), neutrophils, myeloid-derived suppressor cells, cancer stem cells (CSCs), and tumor cells. The base of all interactions forms the extracellular matrix (ECM).

Cancer and host cells undergo complex heterotypic interactions which are crucial for tumor progression and metastasis [78]. Moreover, the environment of tumors also contributes to therapy resistance. It consists of an ECM, stromal cells, and soluble factors. The involved cell types include ECs, infiltrated immune cells like T cells, natural killer cells, macrophages, adipocytes, fibroblasts, and mesenchymal stromal cells (MSCs) (Figure 3) [79]. Stromal contents vary among the different BCa subtypes. About 40–56% of TNBC tumors are defined as stroma-high tumors [80]. In ER^+^ BCa, improved outcomes are associated with increased stromal content, whereas in TNBC a high stromal content predicts poor survival [81].

### 4.1. CAFs

With up to 70% of the whole breast tumor volume, CAFs represent the most abundant cell type in the BCa microenvironment [82,83]. During tissue homoeostasis, fibroblasts are mostly quiescent and play an important role in the organization of matrix proteins including collagens, fibronectin, and elastin. In case of acute inflammation, they are activated as myofibroblasts which are then recruited from the bone marrow to the injured tissue site and take over several processes that are important for wound healing. In response to chronic inflammation or tumor development quiescent tissue sessile fibroblasts are activated, which over time lead to a further disorganization of tissue architecture [84]. As CAFs can disseminate throughout the organism like tumor cells it is suggested that they play an additional role in metastasis [85]. They secrete tumor-promoting mediators, enhance invasion of cancer cells and are able to remodel the ECM to promote tumor growth and metastasis. Recent evidence suggests that CAFs are able to support the renewal of cancer stem cells. Furthermore, CAFs enhance the immunosuppression in the tumor microenvironment and also angiogenesis through the release of cytokines [86]. Recent studies demonstrated that stroma-high tumors correlate with a worse prognosis in BCa patients [87]. Thus, identification of CAF associated antigens would give rise to new opportunities in targeting BCa. The most promising candidate is the fibroblast activation protein (FAP) which is a cell-surface serine protease and highly upregulated in CAFs in BCa and only insignificantly or not expressed at all in adult normal tissues [36,88]. It is known that FAP inhibition can decelerate tumor growth [89]. Therefore, FAP-targeting represents a promising therapeutic approach for BCa.

In 2018, Loktev et al. developed small-molecule radiopharmaceuticals based on a FAP-specific inhibitor. In vitro studies demonstrated highly affine binding of [^125^I]I-FAPI-01 to human and murine FAP-expressing cells with subsequent rapid tracer internalization. Unfortunately, due to enzymatic deiodination the intracellular [^125^I]I-FAPI-01 accumulation decreased after longer incubation times. Therefore, to improve tracer stability a derivative FAPI-02 was coupled to the chelator DOTA for radiolabeling with either ^177^Lu or ^68^Ga, which enables its application as a theranostic tracer. [^177^Lu]Lu-FAPI-02 showed a high binding affinity and rapid uptake in vitro. Moreover, the cellular uptake and retention of [^177^Lu]Lu-FAPI-02 was significantly higher than that of [^125^I]I-FAPI-01. PET imaging with [^68^Ga]Ga-FAPI-02 demonstrated efficient tracer accumulation in FAP expressing fibrosarcoma tumor xenografts and its fast elimination from the blood. In a metastasized breast cancer patient, [^68^Ga]Ga-FAPI-02 not only accumulated efficiently in the primary tumor but also in lymph node and bone metastases [36].

More recently, additional FAPI derivatives (03-15) were published and compared regarding their stability, binding, and biokinetics [37]. The most promising candidate [^68^Ga]Ga-FAPI-04 showed high binding affinity and intracellular retention in vitro, and high tumor uptake in vivo. In a clinical study in patients with metastasized breast cancer [^68^Ga]Ga-FAPI-04 PET/CT delivered high-contrast images with efficient tracer accumulation in metastases and very low uptake in normal tissue. In a theranostic approach, [^90^Y]-FAPI-04 SPECT analysis confirmed tracer accumulation in the metastases. Importantly, treatment with [^90^Y]-FAPI-04 resulted in a significant reduction in pain medication with no side effects [37].

The development of the previously described quinoline-based PET tracers addressing FAP yielded promising results thus [^68^Ga]Ga-FAPI-04 PET/CT was chosen to identify various primary and metastatic cancer types in humans. To accomplish this goal, 80 patients with 28 different tumor entities were included. The highest SUV_max_ was found in lung, breast, and esophageal cancer, cholangiocellular carcinoma, and sarcoma. Interestingly, the tracer was also able to identify liver metastasis as small as 1 cm in diameter. All patients tolerated the examination well and no adverse side effects were observed. The data of this study are clearly promising and may open new applications for noninvasive tumor characterization via FAP-targeting approaches [90].

A recent preclinical study introduced a novel radiolabeled FAP-ligand (FL) [^99m^Tc]Tc-FL-L3 for imaging of TNBC. The SPECT analysis visualized tracer accumulation in the solid tumors. A co-injection of cold FL excess blocked the tumor uptake which proofed the FAP-mediated binding and retention of the radiotracer [38].

### 4.2. TAMs

Inflammation is one of the hallmarks of cancer progression. Here, TAMs play an important role in promoting tumor growth through pro-angiogenic behavior, inhibiting the antitumor immune response and remodeling the ECM [91,92]. Moreover, a high TAM infiltrate density in the primary breast tumor correlates with a worse prognosis [93]. TAMs are the most abundant immune-related cells in the tumor stroma. Cancer cells that underwent EMT are known to activate macrophages to a TAM-like phenotype which in turn produce chemokines to again induce EMT in cancer cells forming a positive feedback-loop [94].

Macrophages are phagocytic immune cells which are involved in tissue homoeostasis, defense mechanisms, and wound healing. Due to microenvironmental stimuli they are polarized to form a heterogeneous population with diverse functions and characteristics. They are subdivided in proinflammatory M1 or anti-inflammatory M2 macrophages. M1 macrophages secrete reactive oxygen species (ROS) and cytokines and are therefore termed as “fight” macrophages. M2 macrophages repair and remodel injured tissue and are involved in debris scavenging and immune modulation. In cancer they act pro-angiogenic by secreting adrenomedullin and VEGFs and suppress the immune response in favor of the tumor [95].

Previous studies have shown that a high number of M2 TAMs in tumor entails chemoresistance and radioprotective effects leading to therapy failure [96]. Targeting TAMs therefore is not only advantageous in inhibiting angiogenesis but also has potential to improve therapeutic efficacy by preventing development of chemoresistance. Up to date several TAM markers for M2 macrophages are known including the mannose receptor C type 1 CD206 and the macrophage scavenger receptors CD204, and CD163 [97].

One common tracer is [^99m^Tc]Tc-tilmanocept which is already approved by the U.S. Food and Drug Administration for lymphatic mapping and sentinel lymph node localization in different malignancies including BCa. The agent binds to the CD206 receptor on the surface of TAMs and dendritic cells which are numerously present in lymphatic tissue resulting in a strong tracer accumulation in this area [98]. Different clinical trials demonstrated that [^99m^Tc]Tc-tilmanocept is superior to [^99m^Tc]Tc-sulfur colloid (SC) due to a faster injection site clearance while primary sentinel node uptake was equivalent [39,99,100,101].

Another group developed macrophage mannose receptor (MMR) targeting nanobodies (^99m^Tc-labeled α-MMR Nb). SPECT analysis in mammary carcinoma mouse model demonstrated the potential of α-MMR Nb as MMR specific approach for efficient targeting of TAMs in solid tumors [40].

Addressing TAMs is also possible with tracer targeting the high-density lipoprotein (HDL) which is specific for macrophages. For this, two ^89^Zr-modified reconstituted HDL (rHDL) have been designed as PET tracers and evaluated in an orthotopic breast cancer mouse model. For design of [^89^Zr]Zr-AI-HDL, only the protein component apoA of HDL was labeled whereas for [^89^Zr]Zr-PL-HDL, only the phospholipid load was labeled. Interestingly, the blood half-life of [^89^Zr]Zr-AI-HDL was nearly three times longer than that of [^89^Zr]Zr-PL-HDL. Tumor uptake was high for both tracer and the specificity was additionally confirmed by localization of radiotracer in ionized calcium-binding adapter molecule 1 (Iba1)-positive areas. Ex vivo histological and flow cytometric analyses validated TAMs as the main target for both radiotracers [41]. A recent study from Mason et al. used [^89^Zr]Zr-HDL to monitor the response to immunotherapy. The mice were administered pexidartinib, an inhibitor of colony-stimulating factor 1 receptor (CSF1R), which is overexpressed on TAMs. PET/CT and also ex vivo analyses demonstrated a correlation between macrophage density and [^89^Zr]Zr-HDL accumulation. Unfortunately, the tracer also highly accumulated in the liver which blurred the delineation of hepatic lesions. However, this study clearly evidences the potential of [^89^Zr]Zr-HDL nanoparticles as macrophage-avid PET tracer for detection of early responses to therapies in tumor microenvironmental-guided approaches [102].

### 4.3. ECs

Angiogenesis describes the formation of new blood vessels and is essential for tumor progression and metastasis as it ensures the supply of the tumor with nutrients and oxygen. ECs line the inside of the vessels, are mostly quiescent in adults and proliferate only once every 150 days. As soon as they are involved in tumor angiogenesis they start to exhibit abnormalities like aneuploidy with abnormal centrosomes and are thus termed tumor endothelial cells (TECs) [103]. These abnormalities are also responsible for a higher proliferation rate suggesting that they lack normal cell cycle checkpoints that inhibit mitosis. One of the main angiogenic factors is VEGF which is secreted by tumor cells and causes an increased proliferative response in ECs [104]. Moreover, TECs develop drug resistance. It has been reported that Ang-2 expression is upregulated during anti-VEGF therapy suggesting it as a treatment escape mechanism. TECs isolated from metastatic tumors exhibit a more pro-angiogenic phenotype and an upregulated expression of VEGFR-1, VEGFR-2, and VEGF [103]. For TNBC, VEGF was shown to be highly deregulated [105].

Earlier studies introduced two VEGFR-2 specific tracer [^64^Cu]Cu-DOTA-VEGF_DEE_ and [^64^Cu]Cu-DOTA-VEGF_121_ for PET imaging. The in vitro results demonstrated that [^64^Cu]Cu-DOTA-VEGF_DEE_ had only slightly lower affinity for VEGF-2 and significantly lower binding affinity for VEGF-1 than [^64^Cu]Cu-DOTA-VEGF_121._ µPET analysis in an in vivo study in BCa xenografted mice visualized higher tumoral tracer uptake and lower renal extraction of [^64^Cu]Cu-DOTA-VEGF_DEE_. Thus, [^64^Cu]Cu-DOTA-VEGF_DEE_ PET presents a potential diagnostic tool for cancer patients especially in monitoring of VEGFR-2 targeted therapies [43].

Since 2008 the humanized monoclonal antibody bevacizumab in combination with weekly paclitaxel is approved for HER2^−^ metastatic BCa [106]. The α-VEGF antibody binds to all of the splice isoforms of VEGF-A and is one of the most studied anti-angiogenic molecules [107].

In a clinical feasibility study Gaykema et al. evaluated the potential of [^89^Zr]Zr-bevacizumab for PET imaging in primary BCa showing tumor uptake in 96.1% of 26 breast tumors. The tumor uptake correlated with VEGF-A protein level. Together these findings suggest a valuable role for biological characterization of tumors and for prediction of the effect of VEGF-A-targeting therapeutics [108].

A different approach targeted VEGFR-1 and -2 with ^89^Zr-labeled single-chain (sc) VEGF mutants. Both engineered human VEGF-A based proteins scVR1 and scVR2 had selective specificity and affinity to either VEGFR-1 or -2. ^89^Zr-radiolabeling made them applicable for PET imaging [44].

In a different study the preclinical potential of (*R*)-[^11^C]C-3-Piperidinylethoxy-anilinoquinazoline (PAQ) to monitor anticancer treatments via PET imaging was evaluated. The ligand PAQ is an analog to vandetanib but exhibits a 40 times stronger inhibitory effect for the VEGFR-2. Therefore, the mice were treated with either paclitaxel (PTX) or a murine analog of bevacizumab, B20-4.1.1 alone or in combination. The (*R*)-[^11^C]C-PAQ PET demonstrated a significant decrease of tumor uptake in the mice treated with B20-4.1.1/PTX combination and in mice after monotherapy with B20-4.1.1. This first preclinical study demonstrated potential of (*R*)-[^11^C]C-PAQ to visualize and quantify the anticancer treatment effects in vivo, however, further studies are needed to examine the ability of this tracer for monitoring treatment response in different dosing protocols [42].

Recently, prostate-specific membrane antigen (PSMA) gained increasing attention as a vascular target of tumor angiogenesis for imaging and therapy. This well-known marker of PCa is highly expressed on PCa cells [109]. Additionally, it was also found to be expressed on the neovasculature of other solid cancers such as kidney, bladder, pancreas, sarcoma, melanoma, lung, and breast cancers [110,111]. In normal vascular endothelium PSMA is not expressed. Most recently, Morgenroth et al. demonstrated that PSMA is expressed by both endothelial and epithelial cells of TNBC which enables a dual-targeting approach when using PSMA ligands for theranostic approaches [48]. As shown in this study, the PSMA expressing TNBC cells induced PSMA expression on endothelial cells and their tube formation. These both effects were observed only in tumor media conditioned by TNBC cells. These results were confirmed in further studies where the pro-angiogenic effect on endothelial cells were induced solely by TNBC cells [45,112].

Bandekar et al. evaluated the therapeutic potential of radiolabeled [^225^Ac]Ac-PSMA ligand which induced high cytotoxic effects in PSMA expressing PCa cells and endothelial cells. These in vitro results provide a promising basis for following in vivo studies to gain deeper insights on the potential of liposomal [^225^Ac]Ac-PSMA for antivascular α-radiotherapy [45].

In a case report different BCa patient tissues were analyzed for PSMA expression. IHC revealed that 60% of the patient showed endothelial PSMA-positivity. Higher PSMA expression correlated with higher grade, NST subtype, hormone receptor-negative, HER2 positive, and TNBC tumors. There was no correlation with tumor size or multifocality of disease. Patients with lymph nodes metastases and higher vascular PSMA expression showed a significantly worse survival. In the very first PSMA radionuclide therapy in a female patient with metastatic breast cancer [^177^Lu]Lu-PSMA was shown to accumulate at the primary tumor side and in the metastatic lymph nodes. However, the treatment was aborted after the second cycle due to severe clinical progression of disease [46].

Since PSMA is a very new target in BCa, it still needs to be further evaluated. However, a radiotracer [^68^Ga]Ga-PSMA, which is successfully used for PET diagnostic of PCa, has also been applied for restaging and evaluation of suitable therapy option in metastatic breast carcinoma. The PET images demonstrated intense skeletal uptake with liver metastasis. [^68^Ga]Ga-PSMA-avid metastases may help to select PSMA-expressing tumors for PSMA-guided therapy [113].

In a different study with 19 BCa patients, the radionuclide ^68^Ga was coupled to PSMA-11 [47]. PET analysis of the BCa patients detected 84% of the 81 tumor lesions. SUV values varied significantly from one patient to another and also from one lesion to another reflecting the heterogeneous PSMA expression. These clinical studies clearly demonstrate the potential of PSMA-targeting in BCa [47].

Since in TNBC PSMA was shown to be expressed in two different cellular compartments a recent study evaluated the potential of targeting PSMA on tumor and tumor associated endothelial cells [48]. Interestingly, in addition to high binding on TNBC cells and associated endothelial cells, the binding of [^68^Ga]Ga-PSMA-11 on TNBC cells was further increased under hypoxic conditions, which suggests that PSMA mediates hypoxia tolerance in cancer cells [48,114]. Furthermore, [^177^Lu]Lu-PSMA-617 disrupted the endothelial tube architecture when cultured in TNBC conditioned medium. In vivo, [^68^Ga]Ga-PSMA-11 PET analysis showed high and specific tracer accumulation in TNBC xenograft in correlation with PSMA expression on tumor cells and the associated vasculature [48]. Together, these findings highlight the potential of PSMA as a vascular and tumor cell associated target in imaging and therapy of TNBC.

The mammary gland epithelium is composed of luminal cells which line the ducts and alveoli, and myoepithelial cells which surround the luminal cells and are enclosed by the basement membrane which is a specialized laminin-rich form of the ECM. Integrins are expressed in the mammary epithelial cells attaching them to the ECM. A lack of adhesion results in proliferative stop of the cell and subsequently a special form of apoptosis termed anoikis. Interaction of integrins with the ECM facilitates myoepithelial cells adhesion and intracellular signaling. Integrins and growth factors are able to activate downstream cascades such as tyrosine kinases and the MAP kinase pathway and as suggested the growth factor receptors. In BCa, integrin expression is altered and plays an important role in the remodeling of the ECM, migration of tumor cells and therefore, metastasis. Moreover, integrins are involved in the EMT which is associated with the expression of matrix-metalloproteinase [115]. Importantly, integrins are expressed on various cell types in the tumor microenvironment including ECs, fibroblasts, and the tumor cells themselves [116]. Since the discovery of the integrin family in the early 1980s many studies demonstrated their pivotal role in cell adhesion and signaling functions of tumor cells, ECs, and other types of the tumor microenvironment [117]. An earlier study evaluated the potential of a radiotracer [^99m^Tc]Tc-NC100692 for BCa imaging. The ligand is highly affine to α_v_β_3_ integrin upregulated on the tumor associated ECs. [^99m^Tc]Tc-NC100692 SPECT analysis visualized all lesion ≥10 mm, however, smaller lymph node metastasis (≤5 mm) remained negative [49]. In a different approach, α_v_β_3_ integrin expression in BCa was analyzed with [^18^F]F-Galacto-RGD PET. The PET study demonstrated an elevated but also highly variable α_v_β_3_ integrin expression in primary and metastatic breast cancer predominantly on ECs, and to lower extent on the tumor cells itself. [^18^F]F-Galacto-RGD uptake was observed in all primary tumors and did not correlate with tumor size. Limitations of this study are the small sample size (*n* = 16 patients) and inability to stage lymph node status [50]. Besides antigens expressed either by the cancer cells or by the cancer microenvironment cellular components (TAMs, CAFs, or ECs) the unique tumor tissue physiological markers like hypoxia, pH, reactive oxygen species, or matrix-metalloproteinases present promising targets for future research.

## 5. Conclusions

In the past decades, the supportive role of the tumor microenvironment in diagnostic and treatment of BCa has been intensively documented. Tumor growth and metastasis is dependent on angiogenesis and the interplay of TAMs, CAFs, ECs, and other cell types. With BCa being a very heterogeneous disease, it is becoming increasingly obvious that there is need for individual and targeted therapies. Conventional and experimental therapies have been introduced to this end, however in some subentities of BCa (especially TNBC) the individual survival of the patients has only improved marginally. Using the potential of radiolabeled ligands might further improve the survival rates. In this review we focused on various radiolabeled tracers for different molecular targets especially in the tumor microenvironment that are currently developed and evaluated. Targets like PSMA and integrins play a special extraordinary role in theranostics as the expression on different cell types allows a dual-targeting approach. Targeting e.g., tumor cells and angiogenesis at the same time provides a larger number of potential binding sites for the respective radiotracer. Nevertheless, most targets are specific for one cell type. A combined approach to target different cellular compartments in the tumor would be conceivable for future tracer design. Meanwhile, the role of nuclear medicine in cancer care is indispensable as it holds a tremendous potential for novel theranostic strategies. Imaging tracer which show a high accumulation in the target can be labeled with therapeutic moieties and thus be implicated in individual therapy design. The challenge is to develop a theranostic radiopharmaceutical with high affinity, low unspecific binding, adequate half-life, low cytotoxic off-site effects, and high target-specificity. By combining molecular biology, chemistry and imaging technologies in an attempt to develop specific stroma- and angiogenesis-targeting agents for BCa, not only patient prognosis and therapy but overall life quality could improve significantly.

## Figures and Tables

**Table 1:** Current radiotracers in preclinical or clinical applications in order of appearance. T.a. = therapeutic approach.

Radiotracer	Target	Tumor Entity	Application	Development Phase	Reference	Potential for TNBC Application
[^18^F]FES	ER	ER^+^ BCa	PET	Phase III	NCT01986569	No
[^18^F]4FMFES	ER	ER^+^ BCa	PET	Phase II	[19]	No
[^18^F]FENP	PR	PR^+^ BCa	PET	Failed	[20]	No
[^18^F]FFNP	PR	PR^+^ BCa	PET	Phase II	NCT03212170	No
[^111^In]In-trastuzumab	HER2/neu	HER2^+^ BCa	SPECT	Early Phase I	NCT01445054	No
[^89^Zr]Zr-trastuzumab	HER2/neu	HER2^+^ BCa	PET	Early Phase I	NCT02065609	No
[^64^Cu]Cu-DOTA-trastuzumab	HER2/neu	HER2^+^ BCa	PET	n.a.	NCT01093612	No
[^89^Zr]Zr-pertuzumab	HER2/neu	HER2^+^ BCa	PET	Phase I	NCT03109977	No
[^68^Ga]Ga-HER2-Nanobody	HER2/neu	HER2^+^ BCa	PET	Phase I	[21]	No
[^68^Ga]Ga-ABY-025	HER2/neu	HER2^+^ BCa	PET	Phase II	NCT01858116	No
[^18^F]F-RL-I-5F7	HER2/neu	HER2^+^ BCa	PET	Preclinical	[22]	No
[^18^F]F-RL-I-2Rs15d	HER2/neu	HER2^+^ BCa	PET	Preclinical	[23]	No
[^131^I]I-SGMIB-2Rs15	HER2/neu	HER2^+^ BCa	SPECT (t.a.)	Preclinical	[24]	No
[^225^Ac]Ac-DOTA-Nb	HER2/neu	HER2^+^ BCa	Ex vivo (t.a.)	Preclinical	[25]	No
[^111^In]In-2Rs15d	HER2/neu	HER2^+^ BCa	SPECT Ex vivo (t.a.)	Preclinical	[26]	No
[^225^Ac]Ac-2Rs15d	HER2/neu	HER2^+^ BCa	Ex vivo (t.a.)	Preclinical	[26]	No
Z_HER2:342_-SR-HP1 + [^177^Lu]Lu- HP2	HER2/neu	HER2^+^ BCa	SPECT (t.a.)	Preclinical	[27]	No
[^111^In]In-2g10	uPAR	uPAR^+^ BCa	SPECT	Preclinical	[28]	Yes
[^64^Cu]Cu-NOTA-ALT-836-Fab	TF	TF^+^ BCa	PET	Preclinical	[29]	Yes
[^89^Zr]Zr-DFO-CR011	gpNMB	gpNMB^+^ BCa	PET	Preclinical	[30]	Yes
[^89^Zr]Zr-M9346A	FRα	FRα^+^ BCa	PET	Preclinical	[31]	Yes
[^86^Y]Y-NM600	Lipid rafts	Lipid rafts^+^ BCa	PET	Preclinical	[32]	Yes
[^177^Lu]Lu-NM600	Lipid rafts	Lipid rafts^+^ BCa	T.a.	Preclinical	[32]	Yes
[^18^F]FTT	PARP1	PARP^+^ Cancer	PET	Preclinical Phase I	[33] NCT03846167 NCT03083288 NCT03604315	Yes
[^124^I]I2-PARPi	PARP1	PARP^+^ Cancer	PET	Preclinical	[34]	Yes
[^131^I]I2-PARPi	PARP1	PARP^+^ Cancer	SPECT	Preclinical	[34]	Yes
[^131^I]I-MAPi	PARP1	PARP^+^ Cancer	SPECT (t.a.)	Preclinical	[35]	Yes
[^125^I]I-FAPI-01	FAP (CAFs)	FAP^+^ Cancer	PET	Preclinical	[36]	Yes
[^177^Lu]Lu-FAPI-02	FAP (CAFs)	FAP^+^ Cancer	PET	Preclinical	[36]	Yes
[^68^Ga]Ga-FAPI-02	FAP (CAFs)	FAP^+^ Cancer	PET	Phase 0/I	[36]	Yes
[^68^Ga]Ga-FAPI-04	FAP (CAFs)	FAP^+^ Cancer	PET	Phase 0/I	[37]	Yes
[^90^Y]Y-FAPI-04	FAP (CAFs)	FAP^+^ Cancer	SPECT (t.a.)	Phase 0/I	[37]	Yes
[^99m^Tc]Tc-FL-L3	FAP (CAFs)	FAP^+^ Cancer	PET	Preclinical	[38]	Yes
[^99m^Tc]Tc-tilmanocept	CD206 (TAMs)	CD206^+^ Cancer	SPECT	Approved	[39]	Yes
[^99m^Tc]Tc-SC	CD206 (TAMs)	CD206^+^ Cancer	SPECT	Phase III	NCT01668914	Yes
[^99m^Tc]Tc-α-MMR Nb	CD206 (TAMs)	CD206^+^ Cancer	SPECT	Preclinical	[40]	Yes
[^89^Zr]Zr-AI-HDL	HDL (TAMs)	HDL^+^ Cancer	PET	Preclinical	[41]	Yes
[^89^Zr]Zr-PL-HDL	HDL (TAMs)	HDL^+^ Cancer	PET	Preclinical	[41]	Yes
[^89^Zr]Zr-bevacizumab	VEGF (ECs)	VEGF^+^ Cancer	PET	Early Phase I	NCT01894451	Yes
(*R*)-[^11^C]C-PAQ	VEGFR-2 (ECs)	VEGFR-2^+^ Cancer	PET	Preclinical	[42]	Yes
[^64^Cu]Cu-DOTA-VEGF_DEE_	VEGFR-2 (ECs)	VEGFR-2^+^ Cancer	PET	Preclinical	[43]	Yes
[^64^Cu]Cu-DOTA-VEGF_121_	VEGFR-2 (ECs)	VEGFR-2^+^ Cancer	PET	Preclinical	[43]	Yes
[^89^Zr]Zr-scVR1	VEGFR-1 (ECs)	VEGFR-1^+^ Cancer	PET	Preclinical	[44]	Yes
[^89^Zr]Zr-scVR2	VEGFR-2 (ECs)	VEGFR-2^+^ Cancer	PET	Preclinical	[44]	Yes
[^225^Ac]Ac-PSMA	PSMA (ECs)	PSMA^+^ Cancer	T.a.	Preclinical	[45]	Yes
[^177^Lu]Lu-PSMA	PSMA (ECs)	PSMA^+^ Cancer	T.a.	Phase 0/I	[46]	Yes
[^68^Ga]Ga-PSMA-11	PSMA (ECs)	PSMA^+^ Cancer	PET	Phase 0/I Preclinical	[47,48]	Yes
[^177^Lu]Lu-PSMA-617	PSMA (ECs)	PSMA^+^ Cancer	T.a.	Preclinical	[48]	Yes
[^99m^Tc]Tc-NC100692	Integrins (ECs)	αvβ3 integrin^+^ Cancer	SPECT	Phase 0/I	[49]	Yes
[^18^F]F-Galacto-RGD	Integrins (ECs)	αvβ3 integrin^+^ Cancer	PET	Phase 0/I	[50]	Yes
